# Inflammation-driven epigenetic memory in infertility and chronic endometritis

**DOI:** 10.1007/s00281-026-01093-w

**Published:** 2026-11-13

**Authors:** Ruizhi Chen, Lianghui Diao, Udo R. Markert, Petra Clara Arck

**Affiliations:** 1 Division of Experimental Feto-Maternal Medicine, Department of Obstetrics and Fetal Medicine, University Medical Center Hamburg- Eppendorf, Hamburg, Germany; 2 Hamburg Center for Translational Immunology, University Medical Center Hamburg-Eppendorf, Hamburg, Germany; 3 Shenzhen Key Laboratory of Reproductive Immunology for Peri- implantation, Guangdong Engineering Technology Research Center of Reproductive Immunology for Peri-implantation, Shenzhen Zhongshan Institute for Reproductive Medicine and Genetics, Shenzhen Zhongshan Obstetrics and Gynecology Hospital, Shenzhen, China; 4 Placenta Lab, Department of Obstetrics, Jena University Hospital, Jena, Germany

**Keywords:** infertility, chronic endometritis, inflammation, epigenetic memory, trained immunity, trained functionality

## Abstract

Chronic endometritis (CE) is a persistent, low-grade inflammatory condition of the endometrium, typically caused by microbial infections. CE is often asymptomatic, leading to frequent underdiagnosis. It is frequently identified during the assessment of causes for infertility. Even after resolving the infectious etiology by antibiotic treatment, infertility persists in a number of patients. Emerging evidence from chronic inflammatory diseases affecting epithelial tissues, e.g., in the gut, suggests that inflammatory conditions can induce long-lasting epigenetic changes, e.g., in innate immune cells. Notably, tissues outside the immune system can also retain memory of inflammatory events and environmental insults via epigenetic modifications. This review summarizes how epigenetic changes can affect endometrial function, drawing on current knowledge of CE. It also discusses how inflammation prompts these changes. We propose that persistent epigenetic memory in both immune and non-immune endometrial cells plays a crucial role in sustaining CE-associated adversities, such as infertility. Future research should focus on pinpointing specific epigenetic patterns in CE and exploring how inflammation-induced memory leads to cellular dysfunction in the endometrium. Understanding these mechanisms will advance CE diagnosis and open doors to new treatment options for infertility, focusing on reversing the inflammatory memory retained upon treatment of CE.

## Introduction

Infertility is a significant global health issue, affecting 8–12% reproductive-age couples [1], with female factors contributing to 35–40% cases [2]. Female infertility can arise from various causes, such as ovulatory disorders, tubal issues, uterine factors, and chronic endometritis [3]. Chronic endometritis (CE) is a persistent, low-grade inflammatory condition of the endometrium, usually caused by bacteria. The adverse impact of CE on infertility is primarily attributed to its disruption of endometrial receptivity and embryo implantation. Therapeutically, antibiotic treatment aimed at eliminating the infection has been shown to improve reproductive success. However, numerous studies indicate that a subset of patients with CE continue to experience ongoing inflammation or unfavorable reproductive outcomes despite receiving antibiotics [4–7].

Evidence published to date has mainly aimed to characterize alterations in immune cell populations in CE, including changes in the distribution of macrophages, natural killer (NK) cells, and plasma cells, alongside alterations in the stromal cellular environment and function [8]. Nevertheless, the molecular mechanisms that maintain the chronic inflammatory state and associated endometrial dysfunction, particularly upon antibiotic treatment, remain poorly elucidated.

We introduce the idea that inflammation associated with CE can cause epigenetic changes in endometrial tissues, thereby contributing to ongoing adverse reproductive outcomes, such as infertility. Epigenetic regulation involves heritable yet reversible changes in gene expression without modifying the DNA sequence. These changes include DNA methylation, histone modifications, and chromatin remodeling [9]. Increasing evidence from chronic inflammatory diseases such as inflammatory bowel disease (IBD) and chronic obstructive pulmonary disease (COPD) shows that inflammation can induce long-lasting epigenetic remodeling [10]. Through mechanisms such as trained immunity and broader forms of epigenetic memory, inflammatory signals may persist and amplify even after the initial trigger is removed [11]. This suggests that similar processes may contribute to the pathological persistence of inflammation in CE. Yet epigenetic regulation in CE remains poorly investigated. Under physiological, non-CE conditions in the endometrium, epigenetic mechanisms are essential for cyclic remodeling, decidualization, and the establishment of the receptive state [12–14].

Below, we review the current understanding of CE pathology, the epigenetic control of normal endometrial function, and inflammation-induced chromatin changes. Based on this finding, we propose a mechanistic model, suggesting that persistent epigenetic memory upon CE treatment may contribute to CE chronicity. This could influence immune cell functions and the development of “trained functionality” in stromal and epithelial cells. Subsequently, this inflammation-driven epigenetic state may lead to defective decidualization, reduced receptivity, and infertility persisting in many women, even upon treatment of CE.

## Causes, diagnostics, and treatment of CE

There are two forms of endometritis: acute and chronic. The relationship between acute and chronic endometritis remains unclear. While both conditions involve endometrial inflammation, the major pathogens causing acute endometritis, Chlamydia trachomatis and Neisseria gonorrhoeae, are rarely detected in CE patients [15]. Long-term clinical studies that directly document the progression from acute to chronic endometritis remain lacking.

**Fig. 1:**
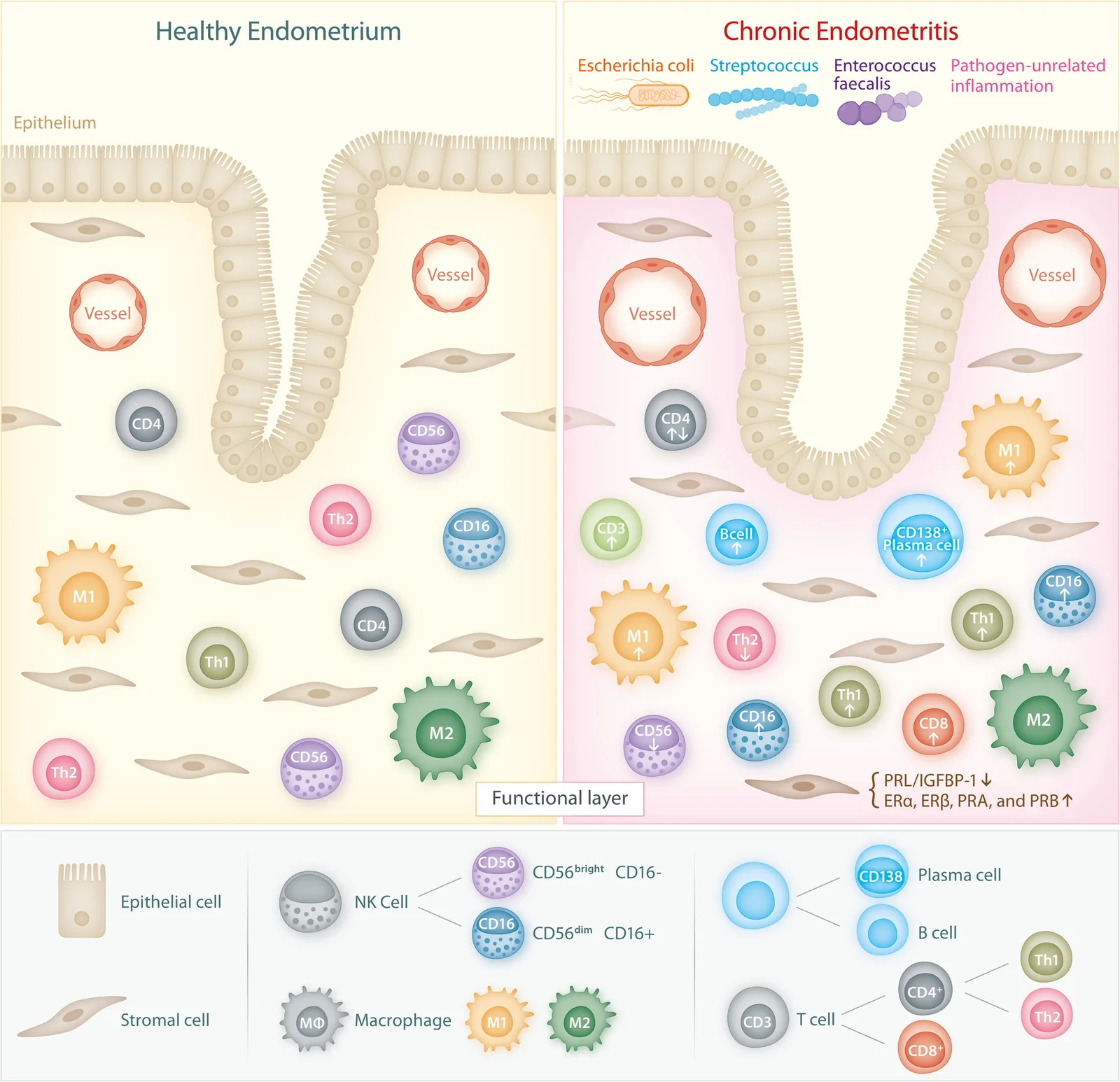
The dysfunctional endometrial microenvironment in CE Characteristics of the immune profile in the healthy endometrium (left) compared to endometrium affected by CE. (right). In the healthy state, the functional layer maintains immune homeostasis, characterized by a predominance of CD56^bright^CD16⁻ uNK cells, a decrease in M1 macrophages, and an increase in Th1/Th2 cells. CE is triggered by persistent inflammatory stimulation associated with microbial infection (e.g., Escherichia coli, Streptococcus, Enterococcus faecalis) or pathogen-unrelated inflammation. CE shows increased infiltration of plasma cells, a shift toward CD56^dim^CD16^+^ uNK cells, M1 macrophage polarization, dysregulated Th1/Th2 subsets, and elevated CD8⁺ cytotoxic T cells. The upward/downwar arrows indicates an ambiguity between studies with regard to up- or downregulation of CD4⁺ T-cell abundance in CE compared with healthy endometrium These immune dysregulations are accompanied by significant structural remodeling, including visible vascular swelling and glandular enlargement. Furthermore, the tissue exhibits pronounced hormonal resistance, marked by an aberrant upward shift in sex steroid receptor expression (ER/PR) and a decreased expression of decidual markers (RPL/IGFBP1). The lower panel provides a legend identifying the cell types and subpopulations depicted in the main illustration

CE is a chronic and persistent inflammatory condition of the endometrium, identified histologically by the presence of plasma cells within the endometrial tissue. Unlike acute endometritis, which is manifested with fever, pelvic pain, and vaginal discharge, CE is often asymptomatic or accompanied by mild symptoms such as abnormal uterine bleeding, pelvic discomfort, dyspareunia, and leukorrhea [15,16]. The etiology of CE is multifactorial, with microbial infection being the primary contributor [17]. Common isolates found in the endometrium of patients with CE include exemplary bacteria such as Streptococcus species, Escherichia coli, and Enterococcus faecalis [16, 18]. Additionally, CE may be associated with pathogen-unrelated inflammatory triggers, such as intrauterine adhesions, multiple endometrial polyps, intrauterine device placement, and endometriosis or autoimmune diseases [19–21]. (Fig. 1) These factors disrupt the endometrial microenvironment, leading to sustained immune dysregulation and impaired tissue homeostasis.

The histopathological characteristics of CE include endometrial surface mucosal edema, separation of epithelial cells and stroma, increased stromal cell density, and plasma cell infiltration in the stroma [22]. Immunohistochemical quantification of CD138^+^ plasma cells is currently regarded as the most reliable and efficient diagnostic method of CE [23, 24]. However, there is no universal consensus on the histopathological definition of CE. This is because CD138 is not exclusively expressed by plasma cells, and the ongoing debate over the correct diagnostic threshold for CD138⁺ cells [25]. A systematic review and meta-analysis assessing various plasma cell thresholds demonstrated that a count of ≥ five CD138⁺ cells per 10 high-power fields (refer to the field of view under the maximum magnification power of the objective being used)is one of the most clinically relevant thresholds associated with adverse pregnancy outcomes [26]. Nevertheless, the presence of low-level CD138⁺ plasma cells in the physiological endometrium makes it difficult to distinguish pathological inflammation from normal immune surveillance [27].

Antibiotic therapy is the main treatment for CE, with doxycycline often serving as the first choice due to its broad-spectrum activity against common bacteria. In most cases, antibiotics successfully reduce or eliminate stromal plasma cell infiltration [27]. For patients who are resistant to initial therapy, alternative or combination regimens are used, alongside additional methods such as intrauterine platelet-rich plasma infusion [28, 29].

## Epidemiology of CE and its impact on reproductive success

Growing evidence shows that CE is closely linked with infertility. Among infertile women, its prevalence ranges from 2.8% to 56.8% [30], with higher rates observed in patients with repeated implantation failure (14%–67.5%) [27, 31–34] and recurrent pregnancy loss (9.3%–67.6%) [33, 35–37]. Antibiotic treatment has demonstrated improvements in reproductive outcomes for women with CE. Specifically, treating chronic endometritis enhances both spontaneous pregnancy rates and live birth rates in patients suffering from unexplained infertility [38]. In women with recurrent pregnancy loss, the live birth rate increased from 7% before therapy to 56% after treatment [39]. Similarly, in IVF or frozen-thawed embryo transfer cycles, CE resolution after antibiotic therapy was associated with significantly higher implantation, clinical pregnancy, and live birth rates compared with women with persistent CE [32, 40, 41]. These studies yet only demonstrate improvement within the CE population and do not directly show that treated CE patients achieve reproductive outcomes equivalent to healthy women.

Nevertheless, the reproductive benefits of antibiotic treatment remain inconsistent across clinical studies. For example, the benefit of antibiotic therapy is less clear in mild CE, defined as one to four CD138 + plasma cells per high-power field, where treatment does not appear to significantly affect pregnancy outcomes [5]. Moreover, a prospective randomized controlled trial showed that oral antibiotics cured over 89% of CE cases but did not significantly improve pregnancy outcomes [77]. Duan et al. also observed a higher incidence of spontaneous abortion in women with cured CE compared to women without CE (11.8% vs. 9.2%) [4].

These apparently inconsistent findings may be partly explained by differences in study design, comparison groups, and reproductive endpoints. Observational studies often compare women with resolved CE with those with persistent CE after treatment, whereas randomized trials assess the effect of antibiotic treatment itself. Importantly, histological resolution of CE does not necessarily guarantee improved reproductive outcomes, and the extent to which endometrial function is fully restored after treatment remains unclear. These findings highlight the complexity of CE and underscore the need to investigate potential mechanisms underlying persistent reproductive impairment in a subset of patients.

## Dysregulation of endometrial immune cells in CE

In a healthy endometrium, innate and adaptive immune cells coexist and are tightly regulated, dynamically contributing to tissue homeostasis and preparing the tissues for possible embryo implantation and the onset of pregnancy. In CE, this finely tuned immune balance is disrupted. B lymphocytes – which account for less than 1% of the leukocytes in healthy endometrium - can reach up to 25% in the endometrium affected by CE [42, 43]. Several adhesion molecules and chemokines involved in recruiting immune cells to the site of infection, such as chemokine (C-X-C motif) ligand (CXCL)1 and CXCL13, are expressed in endothelial and epithelial endometrial cells in CE [44]. This indicates that an abnormal local microenvironment, possibly triggered by bacterial infection, contributes to the selective extravasation of circulating B cells in CE.

Another endometrial immune cell subset influenced by CE is T cells, although reported alterations remain inconsistent across studies. An early flow cytometry study reported an overall increase in endometrial T cells, characterized by elevated CD8⁺ cytotoxic T cells and a relative reduction in CD4⁺ T helper cells [45]. Larger cohort studies confirmed increased uterine infiltration of CD8⁺ T cells and FOXP3⁺ regulatory T cells in CE, while peripheral T cell subsets remained largely unchanged, supporting a localized immune dysregulation within the endometrium [46]. In contrast, other studies reported increased CD4⁺ T cells and an elevated CD4/CD8 ratio in CE, suggesting a distinct immunological phenotype associated with disease severity; however, this analysis was limited by sample size [47]. Together with transcriptomic evidence of immune activation [42, 48], current data indicate a pro-inflammatory endometrial microenvironment in CE, while the direction and magnitude of CD4⁺ T cell changes remain controversial. Such discrepancies may reflect differences in diagnostic criteria, disease severity, and sample size. Some studies have focused on functionally distinct CD4⁺ T-cell subsets, such as T follicular helper (Tfh) cells, which are specialized in supporting B-cell activation, differentiation, and antibody production [49]. Tian et al. showed that Tfh cells increased with CE severity and correlated with a history of miscarriage, suggesting their potential involvement in CE-associated reproductive complications. Earlier studies on CD4⁺ helper subsets reported T helper 1 dominance and reduced T helper 2 in CE [50]. However, both studies were based on small sample size and require validation in larger studies.

Recent findings of ectopic lymphoid aggregates in CE further support a potential interaction between Tfh cells and B cells. These organized immune structures contain germinal-center-B-like cells, proliferating B cells, and Tfh-like cells, suggesting increased local B-cell activation and differentiation. Their greater abundance has also been associated with adverse pregnancy outcomes, underscoring the potential relevance of organized T–B cell interactions in CE-associated reproductive dysfunction [51].

Uterine NK (uNK) cells (CD56^+^CD16^−^) play essential roles in implantation, including regulating trophoblast migration and invasion, promoting spiral artery remodeling, and maintaining tissue homeostasis [52, 53]. Notably, peripheral NK cells typically express CD16 and exhibit stronger cytotoxic activity [54]. Observational studies indicate alterations of uNK cells in CE. Li et al. used genome-wide association study-based Mendelian randomization and found that Human Leukocyte Antigen-DR (HLA-DR)⁺, CD16⁻CD56⁺ NK cells were associated with a lower risk of CE, suggesting a potential protective role; however, this is only indicative and does not reflect actual endometrial cell changes [42]. Chen et al. observed increased CD16 expression in CE endometrial transcriptomes, suggesting a shift toward more cytotoxic NKs, yet functional or protein-level validation is lacking [43]. Flow cytometry studies of endometrial tissue reported decreased proportions of CD56⁺CD16⁻ uNK cells in CE, but larger cohorts are needed to further validate these observations [45]. Other analyses measuring total CD56⁺ NK cells showed no differences [46], suggesting that the overall uNK cell counts may remain stable while their cytotoxic potential changes. These data indicate a possible phenotypic and functional shift in uNK cells in CE, but larger studies with protein-level and functional assays are needed.

Furthermore, multiple studies have reported higher counts of endometrial macrophages in women with CE, especially the pro-inflammatory M1 subtype. Meanwhile, the M2 population seems mostly unaffected [43, 46, 55]. Animal studies further support this observation, showing elevated M1 macrophages in CE model [55]. M1-like macrophages contribute to host defense against microbial infections by producing pro-inflammatory cytokines and antimicrobial mediators. However, sustained inflammatory activation may also contribute to tissue damage and disrupt local immune homeostasis [56]. In CE, high-mobility group box 1 (HMGB1)-induced macrophage pyroptosis has been implicated in amplifying local inflammatory responses [57]. Furthermore, experimental modulation of macrophage polarization toward an M2-like phenotype has been associated with reduced inflammation, improved endometrial repair, and better reproductive outcomes in experimental models of CE [55]. Together, these findings suggest that macrophage activation states may influence both inflammatory responses and tissue recovery in CE, although their precise roles in human disease remain to be fully established.

Overall, CE induces complex and variable immune dysregulation, creating an inflammatory microenvironment that may disrupt maternal-fetal tolerance (Fig. 1). This immune heterogeneity could also help explain why some CE-cured women experience persistent reproductive impairment while others achieve normal fertility.

## Dysfunctional decidualization, aberrant angiogenesis and poor endometrial receptivity in CE

Successful embryo implantation requires both high-quality embryos and a receptive endometrium, which depends on the coordinated regulation of stromal and epithelial cells, orchestrated by hormones, immune cells, and gene expression networks. Decidualization, a process involving the proliferation and differentiation of stromal cells, is essential for establishing endometrial receptivity [58]. In CE, endometrial receptivity and decidualization are both impaired. Endometrial stromal cells from CE patients exhibit reduced expression of decidual markers, such as prolactin (PRL) and insulin-like growth factor-binding protein 1 (IGFBP-1), and show abnormal upregulation of estrogen receptor (ER) α/β and progesterone receptor (PR) A/B [59] (Fig. 1). These defects provide a direct cellular explanation for the clinical observation of implantation failure and early pregnancy loss in CE.

Appropriate angiogenesis is essential for endometrial remodeling and implantation. Carvalho et al. reported several anatomopathological modifications in CE, including increased vascular density, endothelial proliferation, and swelling [60] (Fig. 1). A molecular study further indicates upregulation of hypoxia-inducible factor-1 alpha (HIF-1α), vascular endothelial growth factor A (VEGFA), and vascular endothelial growth factor receptor 2 (VEGFR2) in CE patients, along with increased microvascular density. Antibiotic treatment normalizes HIF-1α and VEGFA expression in cured patients, whereas persistent CE patients show no significant change [61], reflecting a continued dysfunction in vascular regulation.

**Fig. 2:**
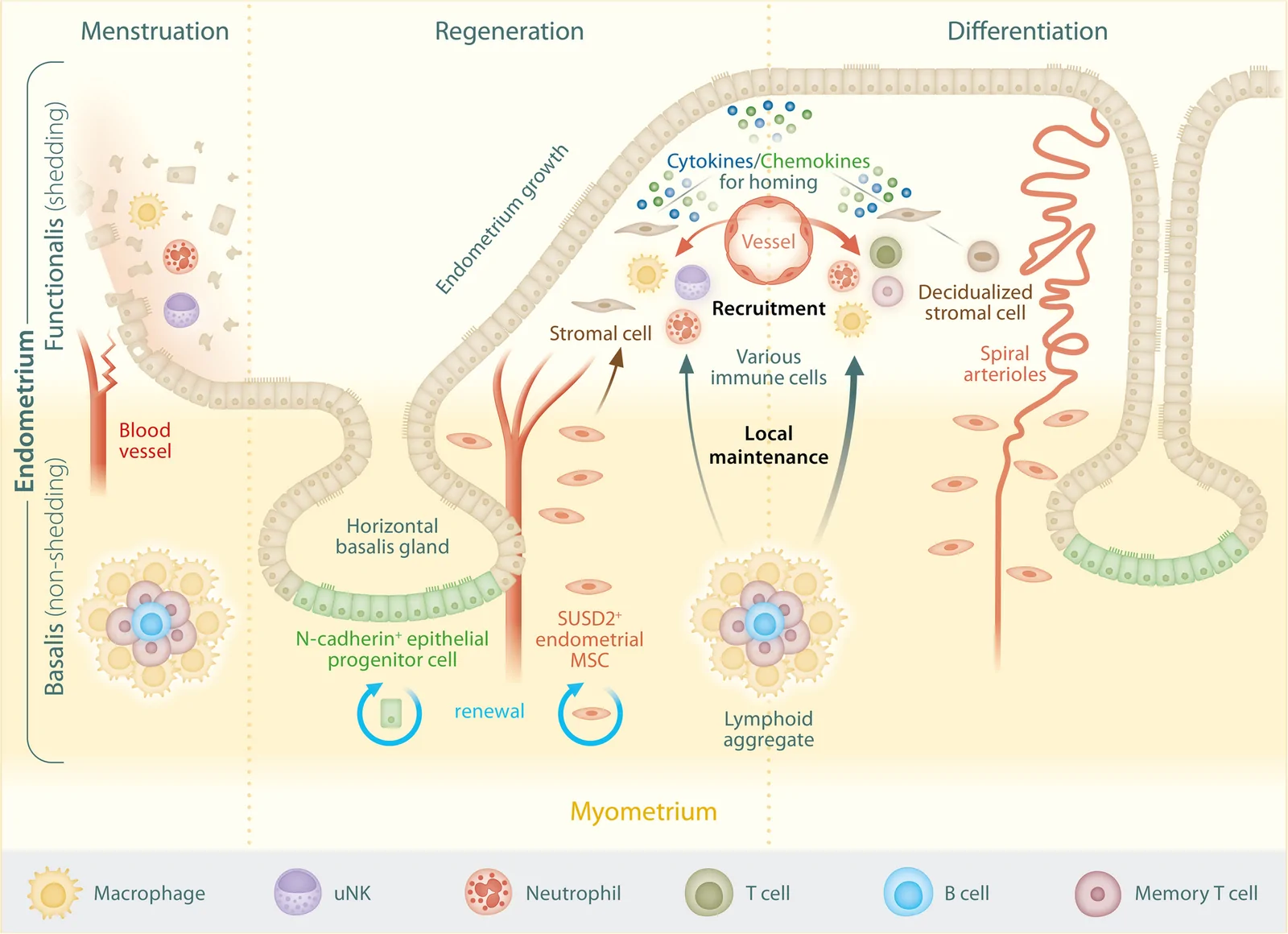
Cyclical regeneration of the human endometrium The human endometrium undergoes cyclical remodeling across menstruation, regeneration, and differentiation. It consists of a superficial functionalis (stratum functionalis) and a basalis (stratum basalis). During menstruation, the functionalis is largely shed, resulting in the loss of differentiated epithelial cells, stromal cells, vasculature, and transient immune cells. In contrast, the basalis is not shedding during menstruation. In consequence, whilst many immune cells within the functionalis are transient, lymphoid aggregates composed of B cells, memory T cells, and macrophages are retained within the basalis. Following menstruation, regeneration of the functionalis is supported by resident stem/progenitor cell populations within the basal niche. Basal epithelial progenitors (e.g., N-cadherin^+^ cells) contribute to the reconstitution of the luminal and glandular compartments [132, 133], while the stromal and vascular components are regenerated by perivascular Sushi domain containing 2 (SUSD2)^+^ endometrial mesenchymal stem cells (MSC) [62, 134]. During regeneration and subsequent differentiation, stromal and epithelial cells produce chemokines and cytokines that guide and attract immune cells from the circulation [65]. During differentiation, stromal cells undergo decidualization, and spiral arterioles form, establishing a receptive endometrial environment. Thus, the basalis serves as a stable cellular reservoir and contributes to the immune maintenance across menstrual cycles [65]

Taken together, impaired decidualization and aberrant angiogenesis constitute key pathological features of CE that compromise endometrial receptivity and implantation (Fig. 1). Notably, CE resolution is commonly assessed histologically by repeating an endometrial biopsy and evaluating CD138⁺ plasma cells after antibiotic treatment. However, it remains unclear whether histological resolution is accompanied by complete restoration of endometrial function. To understand this persistence, one must consider the unique regenerative dynamics of the endometrium (Fig. 2). During menstruation, a large proportion of cells within the endometrium functionalis are discharged, yet the tissue is rapidly reconstructed in subsequent cycles. Current models suggest that this regeneration is supported by cell populations that persist across cycles within the endometrium basalis [62, 63]. The basalis is the permanent layer of the endometrium, located near the myometrium, that is not shed during menstruation. This layer also contains organized lymphoid aggregates, typically consisting of a central cluster of B cells surrounded predominantly by CD8⁺ T cells and an outer layer of macrophages. These structures change in size over the menstrual cycle, suggesting that their organization may be influenced by cyclical hormonal fluctuations [64]. Stromal and epithelial progenitor and stem cells reside in the endometrial basalis and contribute to regeneration of the outer functional endometrial layer throughout the menstrual cycle in preparation for a potential pregnancy [65] (Fig. 2). Therefore, the pathological signatures linked to CE-associated dysfunctions may be retained within cell populations in the basalis. However, the molecular mechanisms underlying these potential persistent alterations remain largely unclear and require further investigation.

## Epigenetic regulation in healthy endometrium

**Fig. 3:**
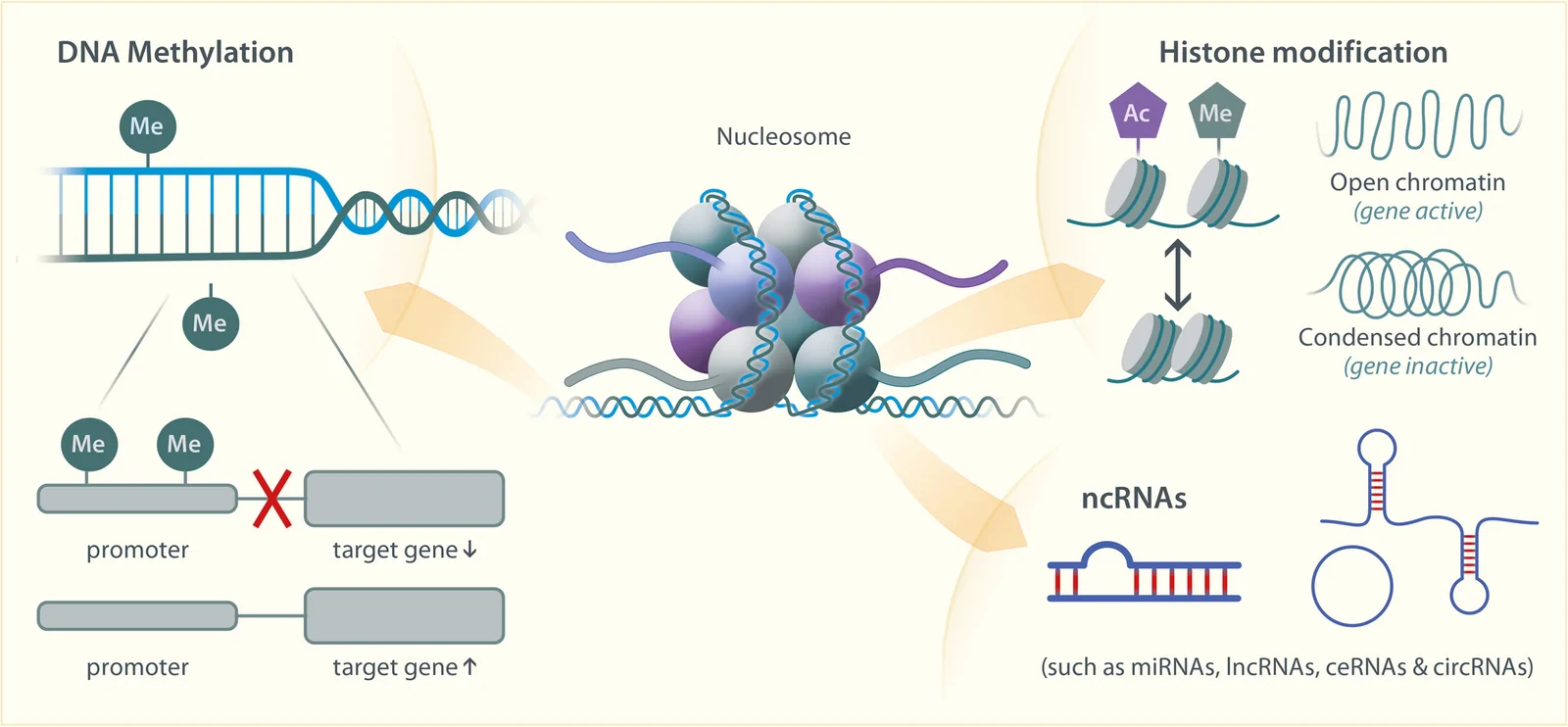
Mechanisms of epigenetic regulation Overview of major epigenetic regulatory mechanisms, including DNA methylation, histone modifications, and non-coding RNAs (ncRNAs). DNA Methylation: The addition of methyl groups (Me) to promoter regions typically acts as a transcriptional repressor. Hypermethylation leads to repressed gene transcription and reduced expression levels, while hypomethylation facilitates an open state for transcriptional machinery, resulting in enhanced expression of the target gene. Histone Modification: The dynamic interplay between acetylation (Ac) and methylation (Me) on histone tails determines the accessibility of the nucleosome. Acetylation typically neutralizes histone positive charges, promoting an open chromatin configuration and upregulating gene expression. In contrast, the impact of histone methylation is site-specific; depending on the particular residue and context, it can either facilitate active transcription or induce condensed chromatin that silences gene activity. Non-coding RNAs: Various ncRNA species, including miRNAs, lncRNAs, ceRNAs, and circRNAs, regulate gene expression post-transcriptionally. These molecules modulate mRNA stability or interfere with translation, typically fine-tuning or suppressing protein synthesis

The endometrium’s ability to undergo complete monthly reconstruction while preserving its unique tissue identity across cycles suggests a profound molecular foundation. Such a process requires mechanisms that can simultaneously drive rapid functional shifts and safeguard stable cellular memory. Epigenetic regulation refers to chemical or physical modifications that affect gene accessibility, thereby regulating how genes are read and expressed, without changing the DNA sequence. Epigenetic regulation involves mechanisms such as DNA methylation, histone modifications, and non-coding RNA-mediated regulation (Fig. 3). These mechanisms can mediate reversible changes in gene expression, some of which may be maintained through cell division [66]. In the healthy human endometrium, which undergoes cyclic changes in response to estradiol and progesterone, epigenetic mechanisms ensure that specific genes are activated at the appropriate time [67].

**Fig. 4:**
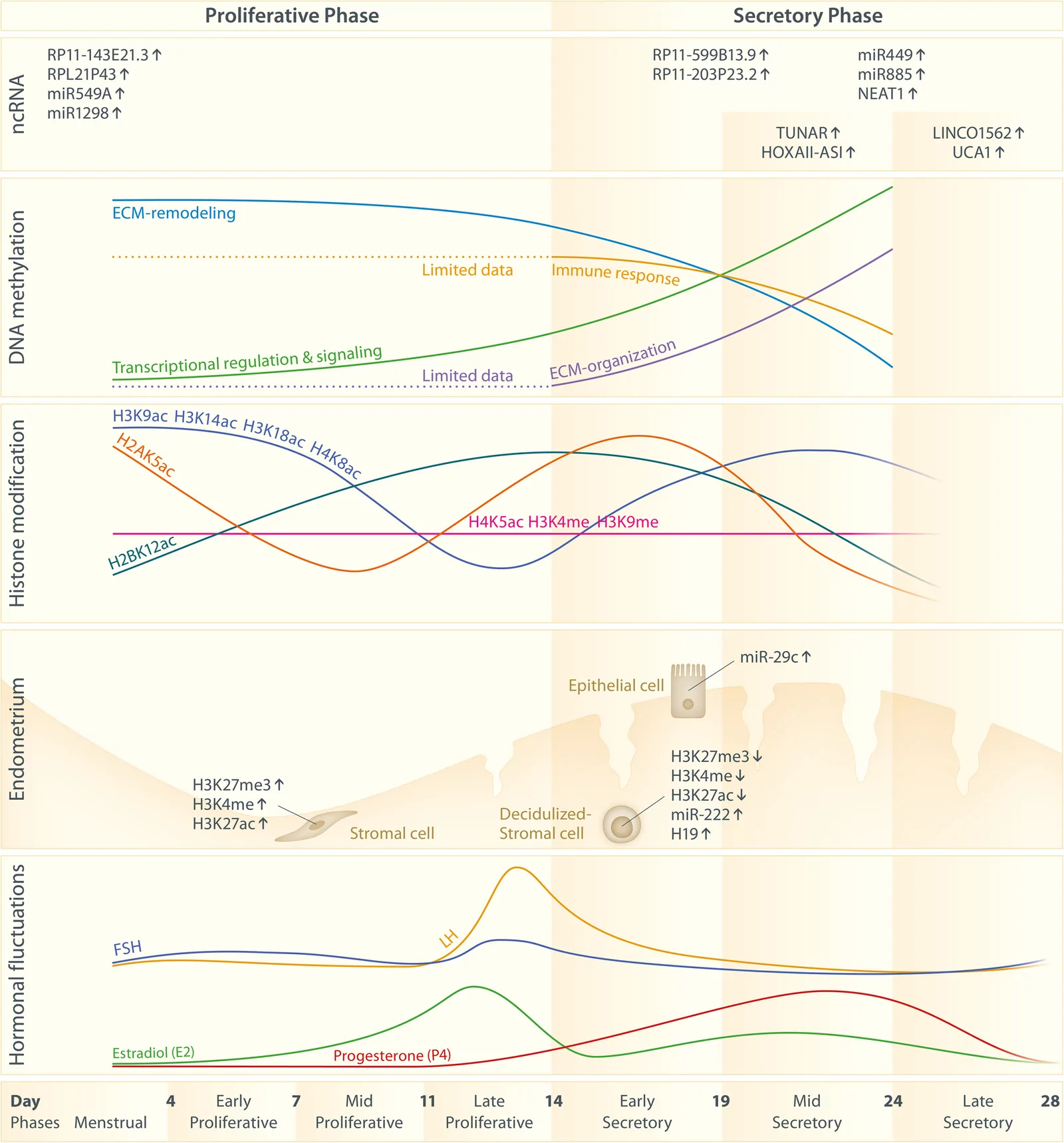
Dynamic epigenetic signatures across the menstrual cycle Trajectories of menstrual cycle-associated epigenetic modifications. Top (ncRNA): Proliferative-phase enriched ncRNAs include RP11-143E21.3, RPL21P43, miR-549 A, and miR-1298, whereas secretory-phase enriched ncRNAs include RP11-599B13.9, RP11-203P23.2, miR-449, miR-885, and Nuclear Enriched Abundant Transcript (NEAT)1 [71]. Specific ncRNAs such as Tcl1 upstream neural differentiation-associated RNA (TUNAR) and Homeobox A11 Antisense RNA (HOXAII-ASIT) increase in the mid-secretory phase, while human genes that encode for a lncRNA, such as LINCO1562 and urothelial cancer-associated (UCA)1, are enriched in the late-secretory phase [72]. Middle panels (DNA methylation and histone modifications): DNA methylation dynamically regulates transcriptional signaling, immune responses, and ECM organization across the cycle. Global histone acetylation marks, including H3K9ac, H3K14/18ac, H4K8ac, H2AK5ac, and H2BK12ac, show cycle-dependent change, while H4K5ac, H3K4me, and H3K9me remain relatively unchanged throughout the menstrual cycle [12, 79]. Bottom panel (cell type–specific regulation): reduced repressive histone marks and enrichment of active chromatin modifications at decidual gene loci. Epithelial cells display secretory-phase–specific miRNA expression, such as increased miR-29c. The hormonal fluctuations panel illustrates the typical relative changes in estradiol (E2) and progesterone (P4) levels, along with the luteinizing hormone (LH) and follicle-stimulating hormone (FSH) surges throughout the menstrual cycle. The x-axis indicates days and phases of the menstrual cycle from menstruation to the late secretory phase

DNA methylation, primarily at the 5-carbon of cytosine in CpG dinucleotides, is a key epigenetic modification. High levels of DNA methylation in gene promoters are frequently associated with transcriptional silencing [68]. In the endometrium, DNA methylation changes dynamically across the menstrual cycle and correlates with gene expression, indicating an important role in steroid hormone–mediated regulation [69]. Genome-wide DNA methylation analyses have shown that the most prominent alterations occur during the transition from the proliferative to the secretory phase, as well as in the late secretory and menstrual stages, indicating that DNA methylation is tightly regulated by cyclical hormonal fluctuation [70, 71]. Functionally, differentially methylated genes are enriched in pathways related to transcriptional regulation and intracellular signaling during the secretory phase. In contrast, genes involved in extracellular matrix (ECM) remodeling and endometrial receptivity are methylated in the proliferative phase [69, 72]. During the transition from the pre-receptive (early secretory) to the receptive (mid-secretory) phase, Kukushkina et al. identified differential DNA methylation patterns associated with changes in gene expression. These methylation-associated transcriptional changes involved genes related to immune responses and extracellular matrix (ECM) organization, suggesting a potential role for DNA methylation in regulating endometrial receptivity [72] (Fig. 4).

Histones are essential components of chromatin’s higher-order structure. Their flexible N-terminal tails can undergo various post-translational modifications, such as acetylation, methylation, and phosphorylation, which regulate chromatin openness and gene activity. These modifications are reversible and are added or removed by specific enzymes known as writers and erasers [74]. Global levels of several histone acetylation marks increase during the early proliferative phase and subsequently decline toward ovulation [12, 75] (Fig. 4). The increase in histone acetylation may be associated with the activation of many genes and pathways necessary for the regeneration of the endometrium’s functional layer. However, further studies are needed to prove this statement. Similarly, an increase in global acetylation levels is observed after ovulation, which could be involved in progesterone-dependent transcriptional activation of secretory-associated pathways required for the differentiation of endometrial glands and stroma [12]. Krusche et al. reported increased HDAC2 protein expression during the secretory phase, suggesting a potential involvement of histone-modifying enzymes in cyclical endometrial regulation, although their direct contribution to changes in histone acetylation remains unclear [76]. This shift in the balance between acetylation and deacetylation may explain the decline of global acetylation in the late secretory phase. Furthermore, several histone modifications are involved in stromal cell differentiation to decidualized stromal cells. In an in vitro decidualized stromal cell model, EZH2, the H3K27 methyltransferase, is downregulated, leading to reduced H3K27me3 at key decidual marker genes such as PRL and IGFBP-1 [77], while H3K27ac and H3K4me are enriched at promoters and distal enhancers of upregulated genes associated with decidualization, such as *WNT4* and *ZBTB16 *[14, 78]. (Fig. 4).

These findings indicate the critical role of histone modification in regulating gene expression during the endometrial cycle. In addition, global and locus-specific changes in histone modifications have been reported in endometriosis and endometrial cancer [79–81]. However, further studies are required to clarify the role of histone modifications in CE.

Non-coding RNAs (ncRNAs), including long non-coding RNAs (lncRNAs), competing endogenous RNAs (ceRNAs), enhancer RNAs (eRNAs), circular RNAs (circRNAs), and microRNAs (miRNAs), have emerged as key regulators of diverse cellular processes [82]. While ncRNAs are genetically encoded, their regulatory effects are epigenetic, as they modulate gene expression without altering the underlying DNA sequence. NcRNAs can modulate gene expression by interacting with nuclear proteins, such as histone-modifying complexes or DNA methyltransferases. Additionally, lncRNAs and miRNAs can directly regulate gene expression by binding to other RNAs, such as mRNA [83]. Although studies on ncRNAs in the normal cyclical endometrium remain limited [84], RNA sequencing analyses have revealed dynamic changes across the menstrual cycle [85, 86]. For example, one of the most studied lncRNAs in the endometrium is H19, which has been detected exclusively in the endometrial stroma. H19 reaches its highest expression during the late secretory phase when progesterone is the dominant hormone. Dysregulation of H19 has been reported in endometriosis and endometrial cancer, where reduced H19 levels are associated with impaired stromal cell proliferation and epithelial-mesenchymal transition process [87, 88].

MicroRNAs (miRNAs) are also dynamically regulated by ovarian steroid hormones throughout the menstrual cycle, particularly during the secretory phase. The different cell types that compose the endometrium express different miRNAs related to endometrial receptivity during the secretory phase. Different endometrial cell types express distinct miRNAs that contribute to endometrial receptivity. For instance, during decidualization, increased miR-222 in stromal cells is associated with reduced proliferation, whereas elevated miR-29c in epithelial cells promotes proper blastocyst attachment [89, 90]. These studies highlight the importance of miRNA functions across the different cell types of the endometrium.

Despite the well-established role of epigenetic regulation in the healthy endometrium, direct evidence of epigenetic alterations in chronic endometritis (CE) remains limited. Available studies have primarily reported dysregulation of non-coding RNAs, including increased expression of miR-27a-3p and miR-124-3p in endometrial tissues from women with CE [91]. More recently, alterations in the metastasis-associated lung adenocarcinoma transcript 1 (MALAT1), miR-142-3p, and phosphoinositide-3-kinase regulatory subunit 1 (PIK3R1) have also been reported in CE [92]. In contrast, the involvement of DNA methylation and histone modifications in CE remains poorly understood. These knowledge gaps underscore the need for further investigation into the epigenetic mechanisms potentially involved in CE pathogenesis.

## Inflammation-induced epigenetic remodeling

Inflammation is not only a transient immune response but also a critical trigger for long-lasting epigenetic alterations. Insights into inflammation-induced epigenetic remodeling have largely emerged from studies in innate immune cells and classical inflammatory disease models. Under chronic inflammatory conditions, sustained immune activation reshapes the activity of chromatin-modifying enzymes through defined transcription factor–dependent pathways, leading to long-lasting changes in histone modifications and chromatin accessibility that form the basis for epigenetic memory across different cell types.

During infection, the pathogen is recognized by pathogen recognition receptors (PRRs), including Toll-like receptors (TLRs) and C-type lectin receptors (CLRs) [93, 94]. Innate immune cells or epithelial cells are rapidly activated through signaling pathways such as Janus kinase–signal transducer and activator of transcription (JAK-STAT), Mitogen-Activated Protein Kinase (MAPK), and nuclear factor κB (NF-κB) [95]. These pathways drive the transcription of cytokines, such as interleukin-6 (IL-6), tumor necrosis factor (TNF, also widely used as TNF-α), and IL-10, and transforming growth factor-β (TGF-β) orchestrates both local and systemic immune responses [96]. This pattern has been extensively characterized across different organs, such as the lung and intestine [97–99]. In human CE, TLR4 expression is markedly increased in endometrial tissues and is accompanied by enhanced activation of MyD88-dependent signaling and multiple levels of the NF-κB cascade, together with elevated TNF-α and IL-1β and reduced IL-10 expression, indicating a sustained pro-inflammatory endometrial microenvironment [100].

Simultaneously, activated transcription factors recruit chromatin-modifying enzymes, including histone acetyltransferases, methyltransferases, and demethylases, to reshape the epigenetic landscape in innate immune cell [101]. Chromatin modifiers contribute to immune regulation through multiple mechanisms. First, chromatin modifiers regulate the expression of both positive and negative regulators of PRR signaling, thereby affecting immune responses. For instance, in mouse macrophages, Ezh1/2-mediated H3K27me3 represses negative regulators such as Tollip and Socs3 [102, 103]. Second, chromatin modifiers regulate the accessibility and structure of chromatin at inflammatory gene loci, facilitating either the amplification or the resolution of inflammation, and may additionally modulate the expression of other genes involved in cellular functions beyond inflammation. For instance, SWI/SNF complexes remodel chromatin at secondary response genes [104], whereas histone demethylases such as Kdm6b remove repressive H3K27me3 marks to enable cytokine transcription [105]. Moreover, cytokines produced during the initial response act in autocrine and paracrine manners, binding to their cognate receptors on the same or neighboring cells. This feedback re-engages the MAPK, JAK–STAT, and NF-κB pathways, reinforcing both cytokine production and chromatin remodeling [101]. These sequential and overlapping signaling-epigenetic events not only shape the magnitude and duration of acute inflammatory responses but also establish longer-lasting chromatin states. The persistence of these states varies markedly across cell populations and is determined by a combination of cellular lineage, the specific inductive stimulus, and the stability of the ensuing epigenetic landscape [106]. Crucially, the durability of these states in the endometrium is tied to the lifespan and regenerative role of the affected cells. While cells in the functionalis are shed, those in the basalis provide a stable reservoir where these chromatin states can be retained across cycles. Despite these insights, it remains largely unexplored whether the persistent pro-inflammatory signaling of the human CE microenvironment is effectively encoded into stable, cell-intrinsic epigenetic remodeling within these basal niches.

## Epigenetic memory: Trained immunity and trained functionality in various tissues

Inflammation can establish long-lasting epigenetic changes that form a cellular memory, influencing subsequent responses. In innate immune cells, these stable modifications underpin long-term functional reprogramming, enhancing responsiveness to secondary challenges, a phenomenon termed trained immunity [107]. Various innate immune cells can acquire such memory after stimulation by microbial components (β-glucan, LPS, BCG), cytokines, or endogenous danger signals [107]. In quiescent myeloid cells, many proinflammatory gene loci are maintained in a repressed chromatin configuration [108], hindering access of the transcriptional machinery to the regulatory regions that drive expression of inflammatory factors [109]. Upon stimulation, innate immune cells acquire epigenetic marks that persist at key promoters and enhancers, reshaping their future responsiveness. For example, the deposition of H3K4me1, H3K4me3, H3K18la, and H3K27ac histone marks in the promoter or enhancer regions of inflammatory response genes leads to permissive chromatin and facilitates expression [106, 110]. MicroRNAs or DNA methylation might also have a role in the induction and regulation of these mechanisms [111, 112]. While inducing trained immunity is protective against infection and other conditions characterized by immunosuppression, such as cancer, excessive or persistent reprogramming of innate immune cells may exacerbate inflammatory responses to exogenous or endogenous triggers, potentially contributing to long-term sterile inflammation and disease [113].

The concept of epigenetic memory extends beyond immune cells. Chronic inflammation can also induce long-lasting not only immune but also functional memory in non-immune cells, a phenomenon we here term “trained functionality”. Similar to innate immune cells, these cells acquire persistent epigenetic changes that influence their future responsiveness. This phenomenon is particularly impactful when it occurs in long-lived structural cells or progenitor populations. For instance, mouse skin epithelial stem cells retain a prolonged inflammatory memory, allowing faster gene activation and barrier restoration upon secondary tissue damage. This memory is maintained through increased chromatin accessibility at key stress-response genes [114]. Furthermore, in Crohn’s disease, patient-derived organoids display persistent hypomethylation at MHC class I loci, linked to altered epithelial-immune interactions [115]. In a murine model of gastrointestinal graft-versus-host disease, intestinal stem cells exhibit durable chromatin and DNA methylation changes that impair regenerative capacity [116]. Studies have reported that fibroblasts are sentinel cells that respond to pathogen [117]. When activated by bacterial infection or cytokines, they enhance inflammatory responses, recruit immune cells, and prolong cytokine signaling through epigenetic modifications [118–120]. Such adaptations can enhance host defense and affect tissue remodeling, but persistent trained functionality in non-immune cells may also drive chronic inflammation and disrupt tissue homeostasis. In summary, these examples show that inflammation-induced epigenetic remodeling establishes long-lasting memory in both immune and non-immune cells, highlighting the dual features of epigenetic memory.

Chronic inflammatory diseases offer compelling evidence that inflammation-induced epigenetic remodeling can shift from an adaptive process to a pathogenic mechanism. Unlike acute inflammation, where chromatin modifications are transient and resolve with the clearance of stimuli, persistent cytokine exposure and repeated activation of inflammatory pathways can stabilize chromatin states, locking cells into hyper-responsive or dysfunctional phenotypes. Research is increasingly and systematically characterizing epigenetic tissue remodeling across chronic inflammatory diseases. Particular attention is being paid to the intestine, lung, and endometrium, which share several tissue characteristics, including epithelial barriers, stromal compartments, and locally regulated immune environments. Insights from diseases of the intestine (IBD) and lung (COPD), for example, now inform approaches to better understand inflammation-associated epigenetic alterations in CE.

IBD includes Crohn’s disease and ulcerative colitis (UC). It is one of the most extensively researched models of chronic mucosal inflammation and, like CE, has been linked to bacterial triggers such as Escherichia coli or Salmonella.

Accumulating evidence demonstrates that long-standing inflammatory stimuli in IBD drive pervasive epigenetic remodeling across multiple cell types within the intestinal niche. Genome-wide methylome analyses have demonstrated that intestinal mucosal cells from IBD patients harbor widespread, stable DNA methylation alterations that affect the epithelial, immune, and stromal compartments [122]. In particular, isolated intestinal epithelial cells from pediatric IBD patients retained distinct methylation and transcriptional signatures, even after ex vivo organoid culture, indicating that some epigenetic changes are cell-intrinsic and persistent rather than secondary to acute inflammation [123]. Beyond DNA methylation, chromatin-level regulation also contributes to this epigenetic memory. In the colon of UC patients and in a mouse model of intestinal inflammation, H3 pan-acetylation and H3K9ac are reduced in the intestinal epithelial cells [124, 125]. This persistent hypo-acetylation likely represents a maladaptive form of epigenetic memory, locking epithelial cells into a repressed transcriptional state that may impair barrier repair or dampen anti-inflammatory responses. Notably, recent evidence indicates that intestinal stem cells from IBD patients also retain an epigenetic memory of prior inflammation, characterized by accessible chromatin landscapes even after extensive ex vivo propagation [126].

COPD is another well-established example in which persistent inflammatory exposure leads to durable epigenetic alterations. While cigarette smoke is the primary environmental driver, recurrent airway colonization or infection with Haemophilus influenzae, Streptococcus pneumoniae, and respiratory syncytial virus (RSV) exacerbates the disease.

Research about epigenetic remodeling in COPD is increasing. Studies have shown that COPD is associated with reduced DNA methylation at CpG promoter regions of inflammation-related genes, such as *IKK* and *NF-κB*, in airway epithelial cells [127]. Parenchymal fibroblasts also display DNA methylation imbalances, which may disrupt tissue repair, accelerate cellular aging, and interfere with inflammatory responses [128]. In alveolar macrophages, globally lower DNA methylation may impair phagocytic function, while increased histone acetylation promotes transcription of pro-inflammatory genes, meaning a decrease of immune tolerance, exacerbating disease progression [129, 130]. Notably, airway epithelial cells from COPD patients and smokers retain persistent functional abnormalities ex vivo, including barrier dysfunction, compromised polarity, and lineage abnormalities, which can persist for up to 10 weeks in long-term culture even in the absence of ongoing smoke exposure [131]. Collectively, these epigenetic alterations across different cell types create a self-reinforcing pathogenic circuit in the lung: hyper-inflammatory immune cells sustain tissue injury. In contrast, epigenetically reprogrammed structural cells exhibit impaired repair and aberrant remodeling.

In summary, these results emphasize that both non-immune and immune cells can develop lasting, cell-intrinsic epigenetic states that are independent of continuous inflammation.

## Inflammation-induced epigenetic changes in CE

**Fig. 5:**
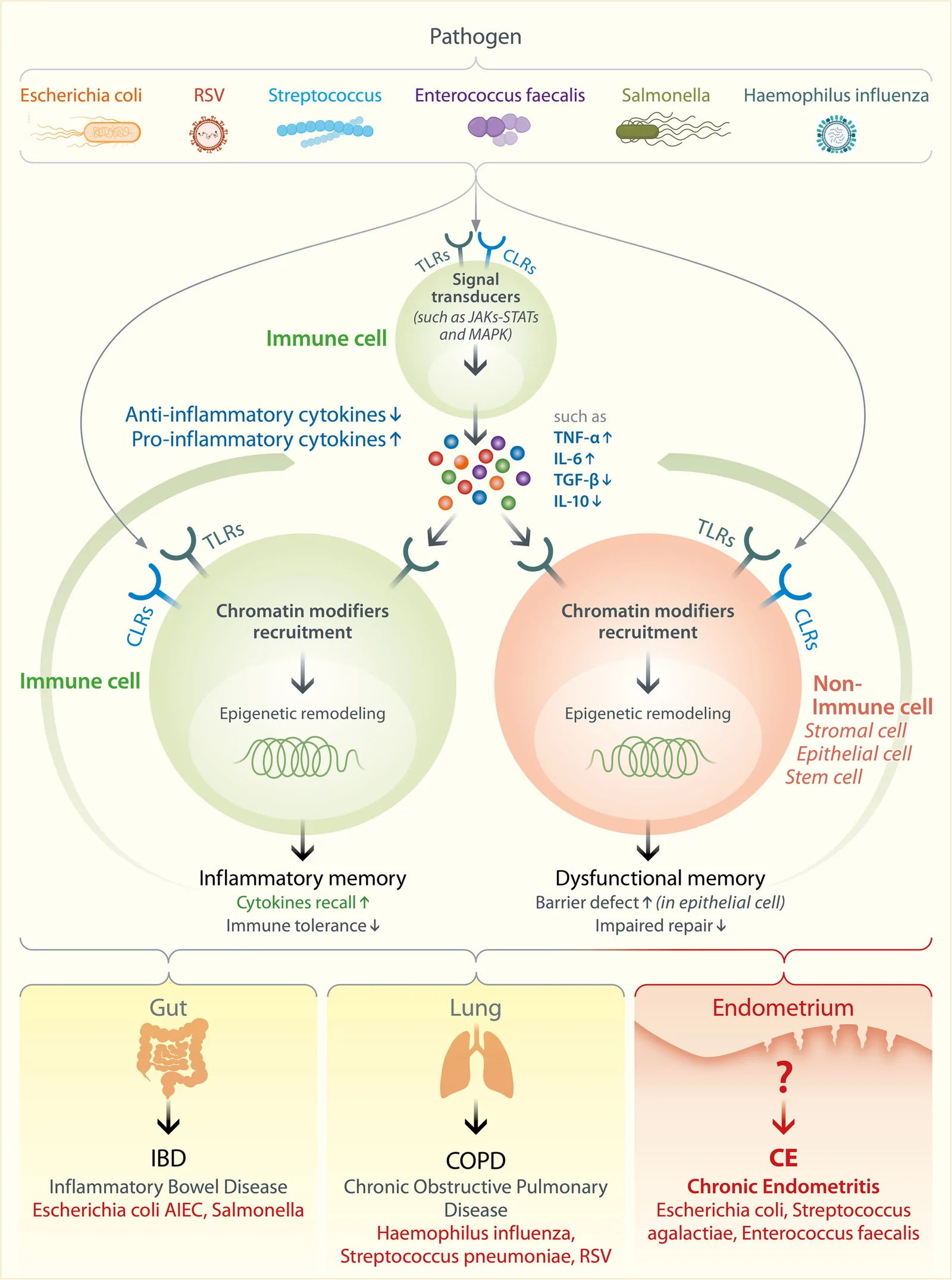
The inflammation-epigenetic remodeling loops in chronic inflammatory diseases Persistent inflammatory stimuli, including pathogen-associated molecular patterns (PAMPs) and damage-associated molecular patterns (DAMPs), activate inflammatory signaling pathways such as JAK–STAT and MAPK, promoting cytokine production and influencing the recruitment and activity of chromatin-modifying enzymes. The resulting epigenetic remodeling may establish persistent cellular alterations, including inflammatory memory in immune cells and dysfunctional memory in non-immune cells, such as epithelial, stromal, and stem cells. These altered cellular states may further reinforce inflammatory signaling and impair tissue repair, establishing a potential self-reinforcing feedback loop. While evidence from inflammatory bowel disease (IBD) and chronic obstructive pulmonary disease (COPD) supports this framework, similar mechanisms in chronic endometritis (CE), particularly their potential persistence in long-lived endometrial cell populations following treatment, remain to be investigated

Findings from IBD and COPD reveal a common pathogenic paradigm in which persistent inflammatory triggers activate canonical signaling pathways, drive cytokine release, and recruit chromatin modifiers. This establishes a self-reinforcing loop in which cytokine-mediated feedback progressively stabilizes altered chromatin states. In immune cells, this process manifests as trained immunity, a stronger and less tolerant immune response. In non-immune cells, such as stromal and epithelial cells, recent findings in IBD have extended this paradigm to intestinal stem cells, resulting in trained functionality, a durable shift in tissue behavior and repair programs (Fig. 5).

This framework may be particularly relevant to CE because of the unique regenerative dynamics of the endometrium. Although much of the functionalis is shed during menstruation, long-lived cell populations within the basalis contribute to tissue regeneration across successive cycles. If chronic inflammation induces stable epigenetic alterations in these cells, these changes could be retained beyond the resolution of detectable inflammation and influence regeneration of the functional layer. This provides a possible link between inflammation-associated epigenetic remodeling and persistent alterations in endometrial receptivity, even after successful antibiotic treatment.

Furthermore, these alterations may persist differently across cell types and individuals, potentially contributing to the heterogeneous reproductive outcomes observed after CE treatment. However, whether such epigenetic memory develops in CE, which cell populations may retain it, and whether it directly contributes to reproductive dysfunction remain important questions for future investigation.

## Conclusion

We suggest that inflammation-induced epigenetic memory may play a role in the chronicity of CE after antibiotic treatment, thereby contributing to the pathogenesis of infertility. This epigenetic memory not only causes trained immunity in endometrial immune cells but also affects stroma cells. The latter may thus experience “trained functionality,” leading to impaired differentiation and poor reproductive outcome. This framework has the potential to enhance CE diagnosis and inspire novel treatments beyond infection management. Future studies should focus on identifying epigenetic markers of CE, investigating how inflammation-triggered memory contributes to cellular dysfunction in the endometrium, and exploring ways to reverse this process.

## Data Availability

No datasets were generated or analysed during the current study.
